# Applying machine learning algorithms to develop a survival prediction model for lung adenocarcinoma based on genes related to fatty acid metabolism

**DOI:** 10.3389/fphar.2023.1260742

**Published:** 2023-10-17

**Authors:** Dan Cong, Yanan Zhao, Wenlong Zhang, Jun Li, Yuansong Bai

**Affiliations:** Department of Oncology and Hematology, China-Japan Union Hospital of Jilin University, Changchun, China

**Keywords:** lung adenocarcinoma, fatty acid metabolism, RiskScore, machine learning, survival probability, prognosis

## Abstract

**Background:** The progression of lung adenocarcinoma (LUAD) may be related to abnormal fatty acid metabolism (FAM). The present study investigated the relationship between FAM-related genes and LUAD prognosis.

**Methods:** LUAD samples from The Cancer Genome Atlas were collected. The scores of FAM-associated pathways from the Kyoto Encyclopedia of Genes and Genomes website were calculated using the single sample gene set enrichment analysis. ConsensusClusterPlus and cumulative distribution function were used to classify molecular subtypes for LUAD. Key genes were obtained using limma package, Cox regression analysis, and six machine learning algorithms (GBM, LASSO, XGBoost, SVM, random forest, and decision trees), and a RiskScore model was established. According to the RiskScore model and clinical features, a nomogram was developed and evaluated for its prediction performance using a calibration curve. Differences in immune abnormalities among patients with different subtypes and RiskScores were analyzed by the Estimation of STromal and Immune cells in MAlignant Tumours using Expression data, CIBERSORT, and single sample gene set enrichment analysis. Patients’ drug sensitivity was predicted by the pRRophetic package in R language.

**Results:** LUAD samples had lower scores of FAM-related pathways. Three molecular subtypes (C1, C2, and C3) were defined. Analysis on differential prognosis showed that the C1 subtype had the most favorable prognosis, followed by the C2 subtype, and the C3 subtype had the worst prognosis. The C3 subtype had lower immune infiltration. A total of 12 key genes (SLC2A1, PKP2, FAM83A, TCN1, MS4A1, CLIC6, UBE2S, RRM2, CDC45, IGF2BP1, ANGPTL4, and CD109) were screened and used to develop a RiskScore model. Survival chance of patients in the high-RiskScore group was significantly lower. The low-RiskScore group showed higher immune score and higher expression of most immune checkpoint genes. Patients with a high RiskScore were more likely to benefit from the six anticancer drugs we screened in this study.

**Conclusion:** We developed a RiskScore model using FAM-related genes to help predict LUAD prognosis and develop new targeted drugs.

## 1 Introduction

Lung adenocarcinoma (LUAD) accounts for about 40% of primary lung tumors. LUAD is one of the tumor types that have rapid metastasis and high mortality, with a survival time shorter than 5 years (Denisenko et al., 2018; Hutchinson et al., 2019; Shi et al., 2022). LUAD, at an early stage, usually has no obvious clinical symptoms and is often diagnosed by adjuvant methods at the middle and late stages or when metastasis occurs (Patz et al., 2014; Skřičková et al., 2018; Sung et al., 2021). Although significant advances have been made in the research and clinical treatment of LUAD, the prognosis of LUAD remains dismal, despite the clinical use of chemoradiotherapy, targeted therapy, and immunotherapy. Currently, the underlying cellular and molecular mechanisms of tumor behavior remain unclear (Lin et al., 2021; Chen et al., 2022a). Therefore, molecular characteristics of LUAD should be comprehensively investigated to improve clinical therapies and the accuracy of prognosis prediction for LUAD.

Lipid metabolism is an important metabolic process for cells. Abnormal fatty acid metabolism (FAM) in cancer cells has been increasingly studied. Carcinogenesis mechanisms of various cancers vary greatly, but they often show similar abnormalities in metabolism. Reprogramming the metabolism of glucose, fatty acids, and other biomolecules could promote the progression of tumor cells (Li and Zhang, 2016). Growing evidence demonstrated that some changes occur in tumor tissues in different processes of FAM (Amiri et al., 2018), including in deciding the types, abundance, and mechanisms of action of lipid-signaling molecules with regulatory functions (Santos and Schulze, 2012). Changes of FAM also affect the proliferation, differentiation, and metastasis of tumor cells (Yu et al., 2018). However, the characteristics and functions of genes related to FAM in LUAD have not been fully explored.

A previous study investigating abnormal FAM showed that the overexpression of fatty acid-binding protein 5 (FABP5) is related to the poor prognosis in LUAD and may be a new clinical target to treat LUAD (Garcia et al., 2022). The downregulation of fatty acid synthase (FASN) interferes with the progression of LUAD through regulating the glucose metabolism and inhibiting the AKT/ERK pathway (Chang et al., 2019). Related drugs could act on the FAM process in LUAD. For example, anlotinib controls LUAD progression through inhibiting FASN-mediated FAM (Shen et al., 2022). Chaoyang Liang et al. also showed that the overexpression of genes related to FAM enzymes (ACOT11) regulates the growth, differentiation, and metastasis of LUAD cells through a variety of signaling pathways (Liang et al., 2020). In addition, Wang et al. developed a fatty acid-related RiskScore model to predict the prognosis of lung cancer patients and identified 38 fatty acid-related genes. Among these 38 genes, eight genes (HGNAT, MCTP2, ENPP5, PLEKHA6, ANKRD29, CNTNAP2, SLC4A5, and ZNF738) have not been reported in previous lung cancer-related studies (Wang et al., 2022a). Therefore, the identification and verification of genes related to FAM may have great potential for developing new prognostic models and improving clinical treatment for LUAD.

In this study, we downloaded genomic information about the clinical characteristics of LUAD from The Cancer Genome Atlas (TCGA) and Gene Expression Omnibus (GEO) databases. Molecular subtypes related to FAM pathways were developed for LUAD, and we further established a risk assessment model based on FAM-related genes using six machine learning algorithms. In addition, we assessed the level of immune cell infiltration and sensitivity to common drugs in different risk groups. The current study provided a better understanding of the mechanism of abnormal FAM in LUAD cells, helping to improve the therapeutic strategies for treating LUAD patients.

## 2 Materials and methods

### 2.1 Data downloading and preprocessing

#### 2.1.1 TCGA-LUAD dataset downloading and preprocessing

Data with clinical phenotypes were obtained from the TCGA database (Liu et al., 2020). Samples without the survival time or state were eliminated to ensure that the survival time of all the included samples was longer than 0 days. Finally, 500 tumor samples and 59 para-cancer tissues samples from the TCGA dataset were obtained.

#### 2.1.2 GEO data download and preprocessing

A set of chip data was obtained from the GEO (Barrett et al., 2013), and the probe was converted into symbol according to the annotation file. Normal tissue samples or those without clinical information were excluded to ensure that the survival time of all the included samples was longer than 0 days, and only LUAD samples were retained through data filtering. Specifically, 289 samples were obtained from the GSE30219 dataset; 226 samples were from the GSE31210 dataset; 196 samples were from the GSE37745 dataset; and 127 samples were from the GSE50081 dataset.

#### 2.1.3 Acquisition of FAM-related genes

From the Kyoto Encyclopedia of Genes and Genomes (KEGG), a collection of 42 related genes was downloaded (Kanehisa and Goto, 2000).

### 2.2 Classification of molecular subtypes

The ConsensusClusterPlus package was used to cluster the TCGA-LUAD and GSE31210 dataset, and the clustering heatmap of the samples was drawn (Liu et al., 2022a). Cumulative distribution function (CDF) was used to obtain the optimal clustering number and relatively stable clustering results. Three molecular subtypes (C1, C2, and C3) were then identified. To further analyze the prognosis of different molecular subtypes, Kaplan–Meier (KM) curves were drawn using the survminer package.

### 2.3 Filtering of differentially expressed genes and enrichment analysis

In order to further screen gene sets related to FAM subtypes, we used the limma package to analyze the differences between C1 and C2+C3, C2 and C1+C3, and C3 and C1+C2 in the TCGA-LUAD dataset under the threshold of | log2 (a Fold Change) | > 1, FDR <0.05 (Ritchie et al., 2015). GO and KEGG enrichment analysis were performed on genes showing abnormal expression using the clusterProfiler software package (Yang et al., 2023).

### 2.4 Construction of the RiskScore model

We used univariate Cox analysis for analyzing the differentially expressed genes (DEGs) (Peng et al., 2021). The prognostic genes with *p* < 0.001 were screened. Machine learning models can be widely used in the medical field due to their excellent performance in predicting classification problems (Choi et al., 2020). Therefore, based on six machine learning algorithms, namely, GBM (Dash et al., 2022), LASSO (Kang et al., 2021), XGBoost (Li et al., 2022a), SVM (Zhou, 2022), random forest (Utkin and Konstantinov, 2022), and decision trees (Streeb et al., 2022), the DEGs in the comparison pairs of C1 and C2+C3, C2 and C1+C3, and C3 and C1+C2 were comprehensively analyzed, and the characteristic genes were obtained by overlapping analysis. A stepwise regression method was used to further compress the characteristic genes. We calculated the β value by multivariate Cox analysis (Zhang et al., 2021a). The calculation formula of the model is as follows:

RiskScore=Σβi×Expi.

In the formula, *Expi* is the expression value of key FAM-related genes and β is the Cox regression coefficient of the key genes.

According to the abovementioned formula, the RiskScore of each TCGA-LUAD sample was determined and then processed by the Z-score (DeVore, 2017). Then, the samples with a RiskScore less than 0 were categorized as the low-RiskScore group, while those with a RiskScore greater than 0 were categorized as the high-RiskScore group. Five sets of chip data (GSE31210 cohort, GSE19188 cohort, GSE30219 cohort, GSE37745 cohort, and GSE50081 cohort) were used to calculate the RiskScore by the same method. A receiver operating characteristic (ROC) curve was obtained using the timeROC package (Lu et al., 2022a).

### 2.5 Comparison of clinical features

The clinicopathological features (Gender, Event, T. Stage, M. Stage, N. Stage, and Stage) in different molecular subtypes and different RiskScore groups in the TCGA cohort were analyzed. The pheatmap package of R software was applied to plot a heatmap to examine the distribution of samples with different clinical features (Zhang et al., 2021b).

### 2.6 Establishment of a nomogram

The relationships between clinical features, RiskScore, and prognosis were assessed applying univariate and multivariate Cox analyses (van de Vijver et al., 2002). The model prediction efficiency was evaluated by developing a nomogram combining key clinicopathological features using the rms package (Liu et al., 2021a). A calibration curve was used to evaluate the predictive power of the model and to test the prediction performance of the nomogram (Van Calster et al., 2019). We also used the ggDCA package to assess the stability of the decision curve analysis (DCA) and to plot the calibration curve and DCA for the nomogram in predicting 1-, 3-, and 5-year prognosis (Van Calster et al., 2018).

### 2.7 Mutation analysis

Data for copy number variant (CNV) were downloaded to compare the deletion or amplification of genes associated with FAM pathways (Wu et al., 2020). Then, mutation data of single nucleotide variants (SNVs) were downloaded and a waterfall map was generated using the maftools package to display SNV mutations in FAM-related pathway genes (Li et al., 2022b).

Differences in genomic changes were examined in different molecular subtypes. Mutated datasets were processed using mutect2 software (Pei et al., 2021). Genes showing a mutation frequency greater than 3 were filtered. Fisher’s test was applied to detect frequently mutated genes in each subtype (*p* < 0.05).

### 2.8 Pathway difference analysis

The FAM pathway scores were calculated by single sample gene set enrichment analysis (ssGSEA) (Zhuang et al., 2021). Differences in FAM-related pathway scores between LUAD and para-cancer tissues were compared by the Wilcoxon signed-rank test (Divine et al., 2013). We used the pheatmap package to draw heatmap to show the expression of related genes (Zheng et al., 2022).

In order to characterize biological process pathways, the GSVA software package (Hänzelmann et al., 2013) was used to analyze all the relevant gene sets in the Hallmark database. The Kruskal test was performed to examine differentially activated pathways in different molecular subtypes (Liu et al., 2021b). Significant pathways were selected under *p* < 0.05, and the heatmap of functional enrichment scores of each subtype was generated.

### 2.9 Comparison of immune abnormalities

Immune infiltration was evaluated by Estimation of STromal and Immune cells in MAlignant Tumors using Expression (ESTIMATE), and the differences in immune scores were compared (Yang et al., 2021). Then, CIBERSORT algorithm was used to calculate the abundance of 22 kinds of immune cells and compare the differences in immune cell scores (Zhang et al., 2022a). A variety of immune cell characteristic genes were identified (Charoentong et al., 2017). We compared the differences of 28 immune cell scores using the ssGSEA. Furthermore, the expression of gene multiple immune checkpoint genes was analyzed between different RiskScore groups (Danilova et al., 2019).

### 2.10 Drug sensitivity analysis

The R language pRRophetic package could be used to predict patient sensitivity to drugs. Several commonly used drugs such as erlotinib, paclitaxel, MG-132, rapamycin, sunitinib, and cisplatin were selected (Skalniak et al., 2013; Landi and Cappuzzo, 2015; Bhaoighill and Dunlop, 2019; Wang et al., 2020; Lu et al., 2022b).

### 2.11 Statistical analysis

This study mainly used R software for statistical analysis. A *p* < 0.05 was defined as a statistically significant difference. The Wilcoxon test was used to assess differences in immune abnormalities between RiskScore groups.

## 3 Results

### 3.1 Abnormal FAM-related pathway genes in LUAD

**FIGURE 1:**
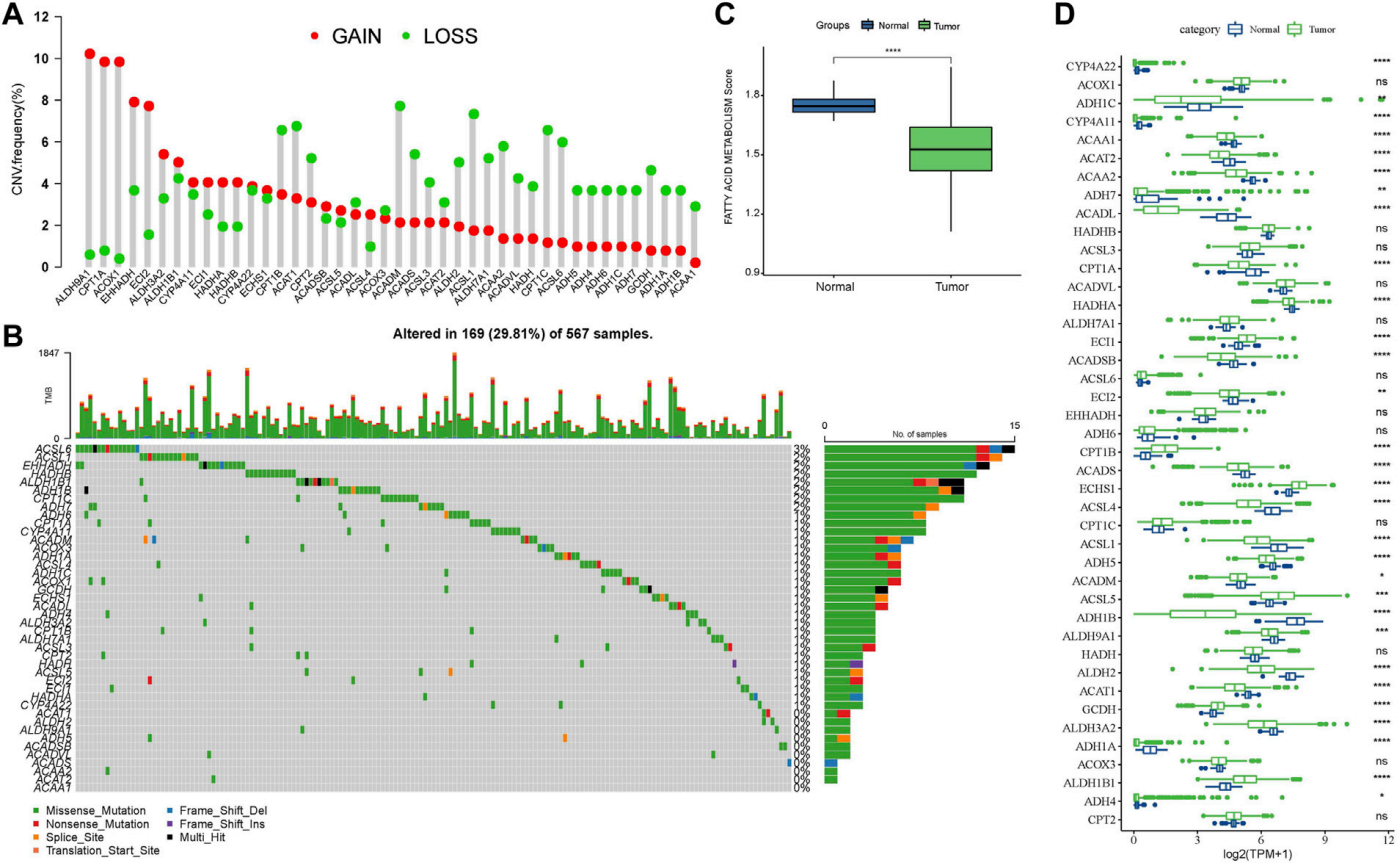
Abnormal FAM-related pathway genes in LUAD. **(A)** Proportion of gene deletion and amplification in the CNV of FAM-related pathway genes. **(B)** Waterfall diagram of mutant information in the SNV of FAM-related pathway genes. **(C)** Comparison of FAM pathway scores in LUAD and para-cancer tissues. **(D)** Comparison of expression of FAM-related pathway genes in LUAD and para-cancer tissues.

After selecting FAM-related pathway genes, we found that some genes, such as ACADM, ACSL1, and CPT1C, tended to show deletion, while some genes, such as ALDH9A1, CPT1A, and ACOX1, tended to show amplification (Figure 1A). The SNV of the genes related to FAM pathways was shown in waterfall diagram, and it was found that ACSL6 had the highest mutation rate and was mostly missense mutation (Figure 1B). A comparison of scores of FAM pathways showed that LUAD tissues had a lower score of FAM pathways compared to para-cancer tissues (Figure 1C). The expression of 24 FAM-related pathway genes was more active in para-cancer tissues than that in LUAD tissues, such as ACAA1, ACAT2, and ADH1B (Figure 1D). These results suggested that FAM-related genes may have an impact on the progression of LUAD.

### 3.2 Classification of molecular subtypes based on genes related to FAM pathways

**FIGURE 2:**
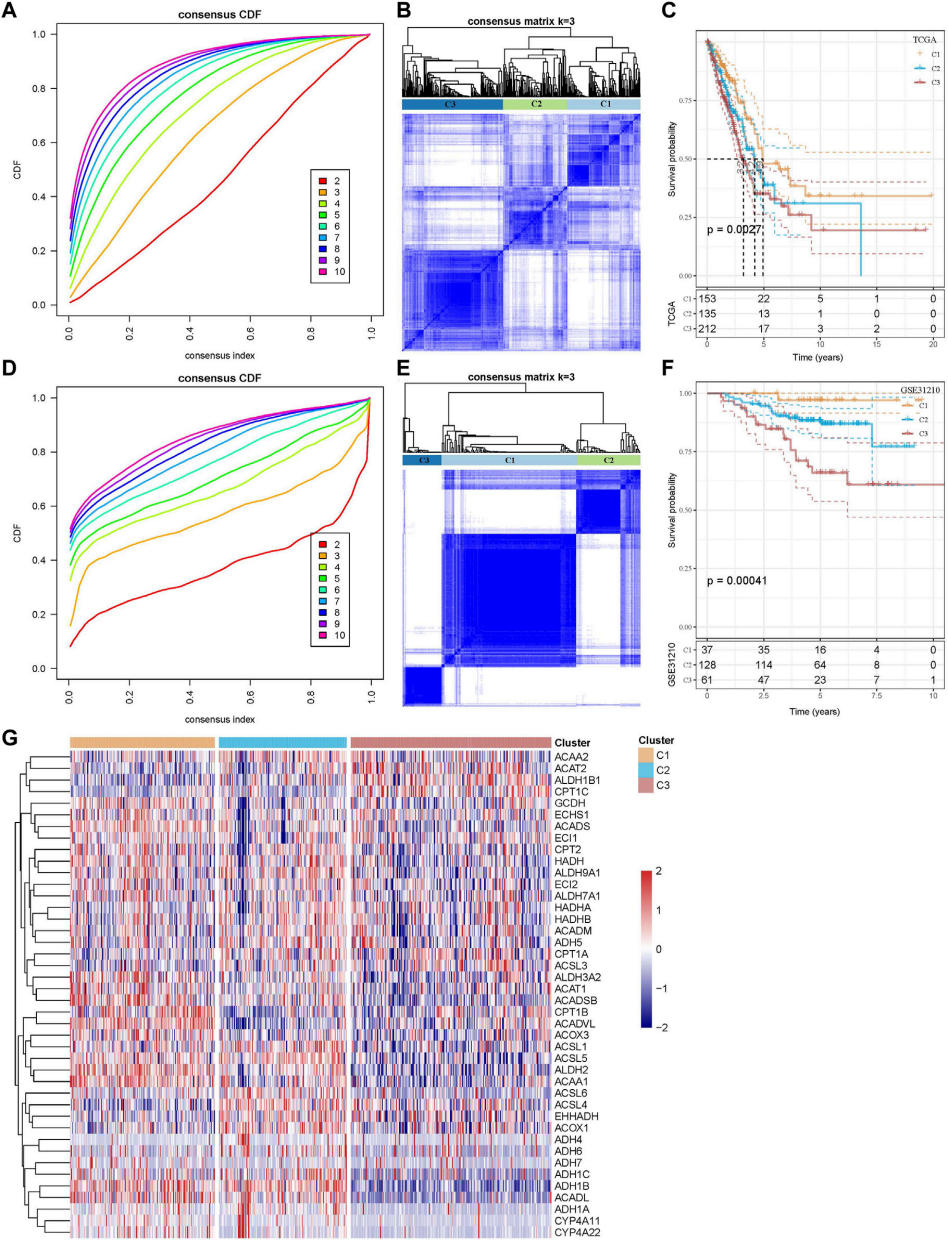
Construction of molecular subtypes based on genes related to FAM pathways. **(A)** CDF curve of TCGA cohort samples. **(B)** Clustering heatmap of samples in the TCGA cohort when consensus k = 3. **(C)** Relationship between the prognoses of three TCGA subtypes is shown by the K–M curve. **(D)** CDF curve of GSE31210 cohort samples. **(E)** Clustering heatmap of samples with consensus k = 3 in the GSE31210 cohort. **(F)** K–M curve of the relationship between the prognoses of three subtypes of GSE31210. **(G)** Heatmap of the expression of FAM-related pathway genes between three subtypes in the TCGA cohort.

In the TCGA dataset, the CDF curve showed that cluster 3 was a relatively stable clustering (Figure 2A). Finally, three molecular subtypes of C1, C2, and C3 were defined based on the sample clustering heatmap (Figure 2B). Further analysis of the K–M curves for the three molecular subtypes showed significant differences in terms of prognostic survival among the three subtypes (*p* = 0.0027). Overall, C1 had the best survival outcome, followed by C2 and C3 (Figure 2C).

We used the abovementioned methods to analyze and classify the GSE31210 dataset. The CDF results showed that cluster 3 also had relatively stable clustering results (Figure 2D). At k = 3, the three molecular subtypes were significantly different (Figure 2E). There were also significant differences in prognostic survival among the three subtypes (*p* = 0.00041). The survival of C1 was found to be the most favorable, while that of C3 was the worst, and the overall results were similar to those of the TCGA dataset (Figure 2F).

The gene expression of FAM-related pathways in the three subtypes was shown in the heatmap. It was found that the gene expression of FAM-related pathways in C1 was relatively active, while that in C3 was relatively poor (Figure 2G). These results suggested that FAM-related subtypes were associated with different prognosis, and that tumors of different subtypes had large differences in the FAM status.

### 3.3 Significant differences in clinicopathological characteristics among the three subtypes

**FIGURE 3:**
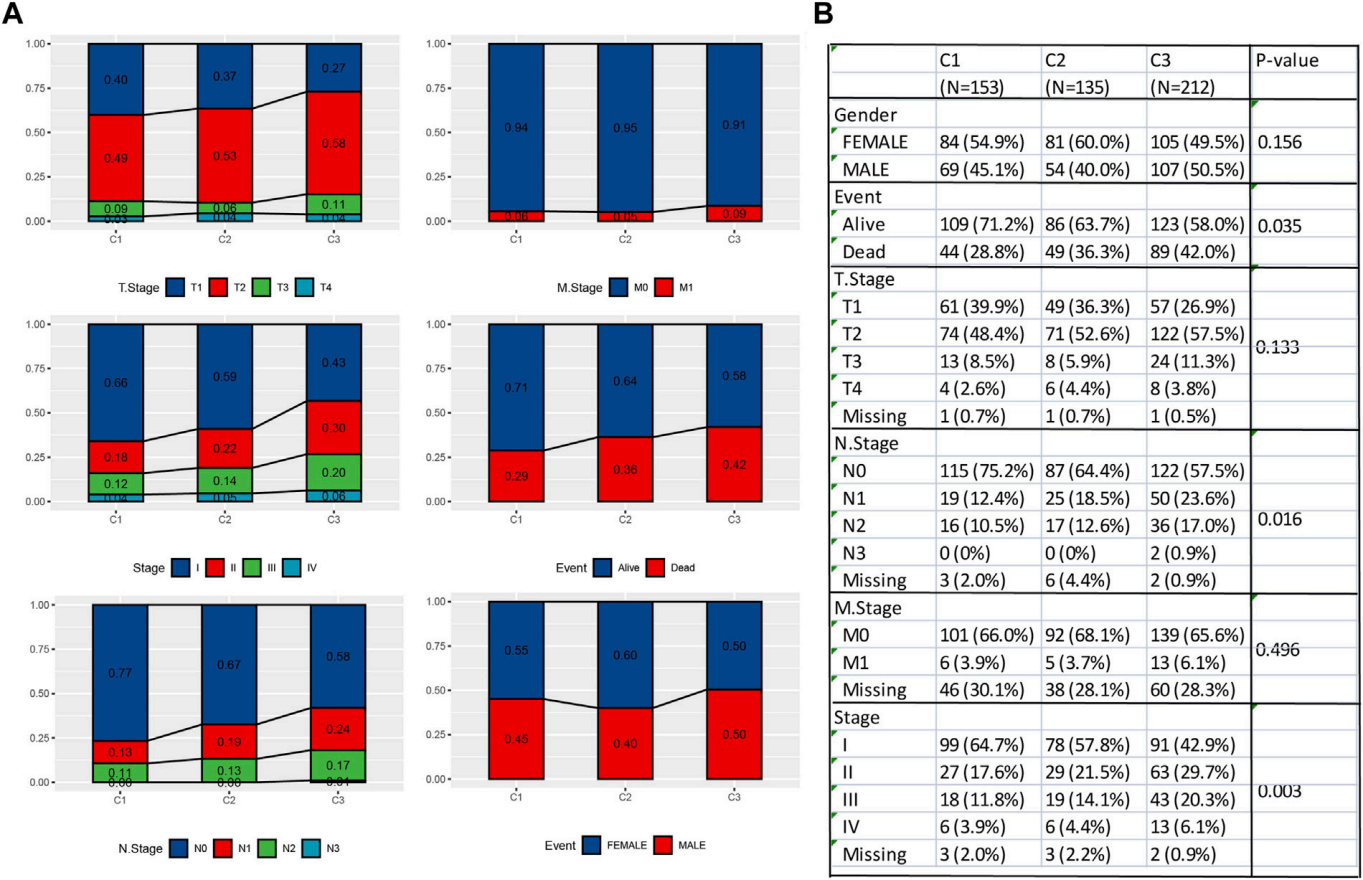
Differences in clinical characteristics among molecular subtypes. **(A)** Bar chart of pair-to-pair comparison of three molecular subtypes in the TCGA cohort. **(B)** Comparative table of each clinical characteristic molecular subtype.

The clinicopathological characteristics of different subtypes in the TCGA cohort were analyzed. There were significant differences in three clinical indicators (Event, N Stage, and Stage) of the three subtypes (*p* < 0.05). Event showed that C1 had a significantly higher survival probability compared to C2 and C3, while C3 had the worst survival outcomes. As for N stage, C1 had the highest proportion of N0, while C3 had the highest proportion of N2. In Stage, C1 had the largest proportion in stage I, while C3 had the largest proportion in stage IV. Therefore, the outcome, clinical grade, and staging of C1 were relatively favorable, while those of C3 were unfavorable (Figure 3).

### 3.4 Mutant characteristics and differential activation pathways of the three subtypes

**FIGURE 4:**
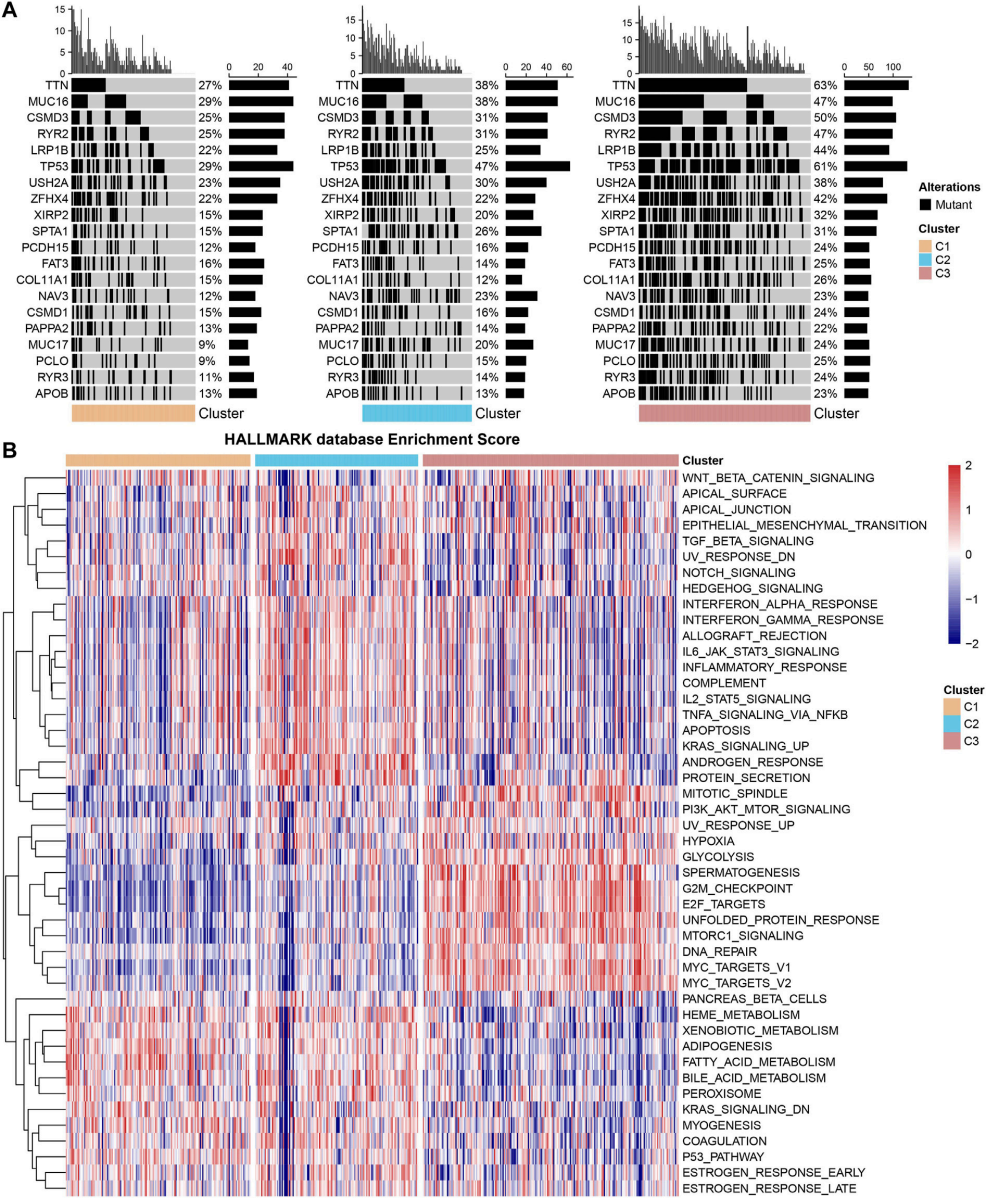
Mutant characteristics and differential activation pathways of molecular subtypes. **(A)** Different molecular subtypes in the TCGA cohort were analyzed by somatic mutation analysis. **(B)** Heatmap of functional enrichment scores of each subtype in the TCGA cohort.

Differences in genomic changes among subtypes in the TCGA cohort were analyzed. The mutation dataset of TCGA was processed by mutect2 software, and a total of 9,922 genes were screened. Fisher’s test was used for screening with *p* < 0.05, which filtered 770 genes. The top 20 genes were selected for further analysis on the characteristics of somatic mutations. The results showed that C1 had the lowest mutation rate and C3 had the highest mutation rate (Figure 4A). Moreover, whether differentially activated pathways were present in different subtypes were explored. Some screened pathways were found to be significantly differentially activated in different subtypes, for example, PI3K AKT MTOR SIGNALING, G2M CHECKPOINT, and FATTY ACID METABOLISM. The FATTY ACID METABOLISM pathway was actively expressed in the C1 and C2 subtypes but less expressed in the C3 subtype (Figure 4B). This could explain a poorer prognosis of C3.

### 3.5 Analysis of immune abnormalities of subtypes

**FIGURE 5:**
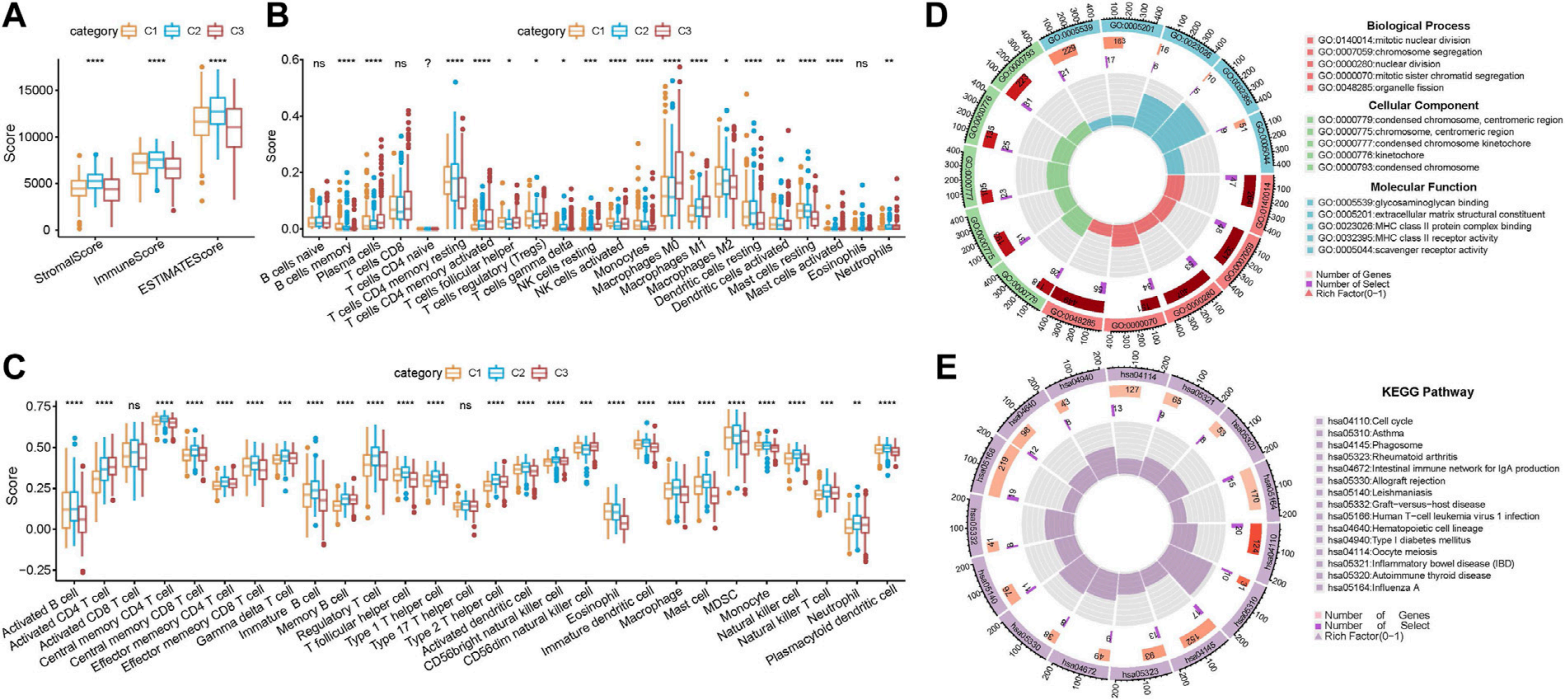
Analysis of immune abnormalities of molecular subtypes and enrichment analysis of differential genes. **(A)** Comparison of immune scores among different subtypes. **(B)** Comparison of 22 immune cell scores among different subtypes. **(C)** Comparison of the abundance of 28 immune cells among different subtypes; * *p* < 0.05, ** *p* < 0.01, *** *p* < 0.001, **** *p* < 0.0001, and ns represents *p* > 0.05. **(D)** Circle diagram of the top five functional enrichment analyses of differential genes GO enrichment analysis. **(E)** Circle diagram of the top 15 functional enrichment analyses for KEGG enrichment analysis of differential genes.

Analysis on differences in the immune microenvironment among the three subtypes showed that C3, with a poor prognosis, had the lowest scores of StromalScore, ImmuneScore, and ESTIMATEScore, which indicated lower immune infiltration of C3 (Figure 5A). The abundance of 18 kinds of immune cells, such as B-cell memory, was different among the three subtypes (Figure 5B). The calculation results of 28 immune cell scores, such as activated B cells and activated CD4 T cells, demonstrated differences in 26 immune cell scores among the three subtypes (Figure 5C).

### 3.6 Screening and enrichment of FAM-related genes

To screen gene sets associated with FAM subtypes, differential analysis was performed for C1 and C1+C2, C2 and C1+C3, and C3 and C1+C2. A total of 124 upregulated genes and 123 downregulated genes were screened in the comparison between C1 and C2+C3; 59 upregulated genes and four downregulated genes were screened in the comparison between C2 and C1+C3; and 160 upregulated genes and 276 downregulated genes were screened in the comparison between C3 and C1+C2. There were 493 DEGs in total. Circle diagrams of GO enrichment analysis on the top five functional enrichment analyses were plotted, and we observed that mitotic sister chromatid segregation was the most significant biological process. The condensed chromosome centromeric region was the most prominent cellular component. The MHC class II receptor activity was the most active molecular function (Figure 5D). Asthma was found to be the most significant pathway in the circle diagram of the top 15 functional enrichment analyses on the differential genes (Figure 5E).

### 3.7 Construction and evaluation of a RiskScore model

**FIGURE 6:**
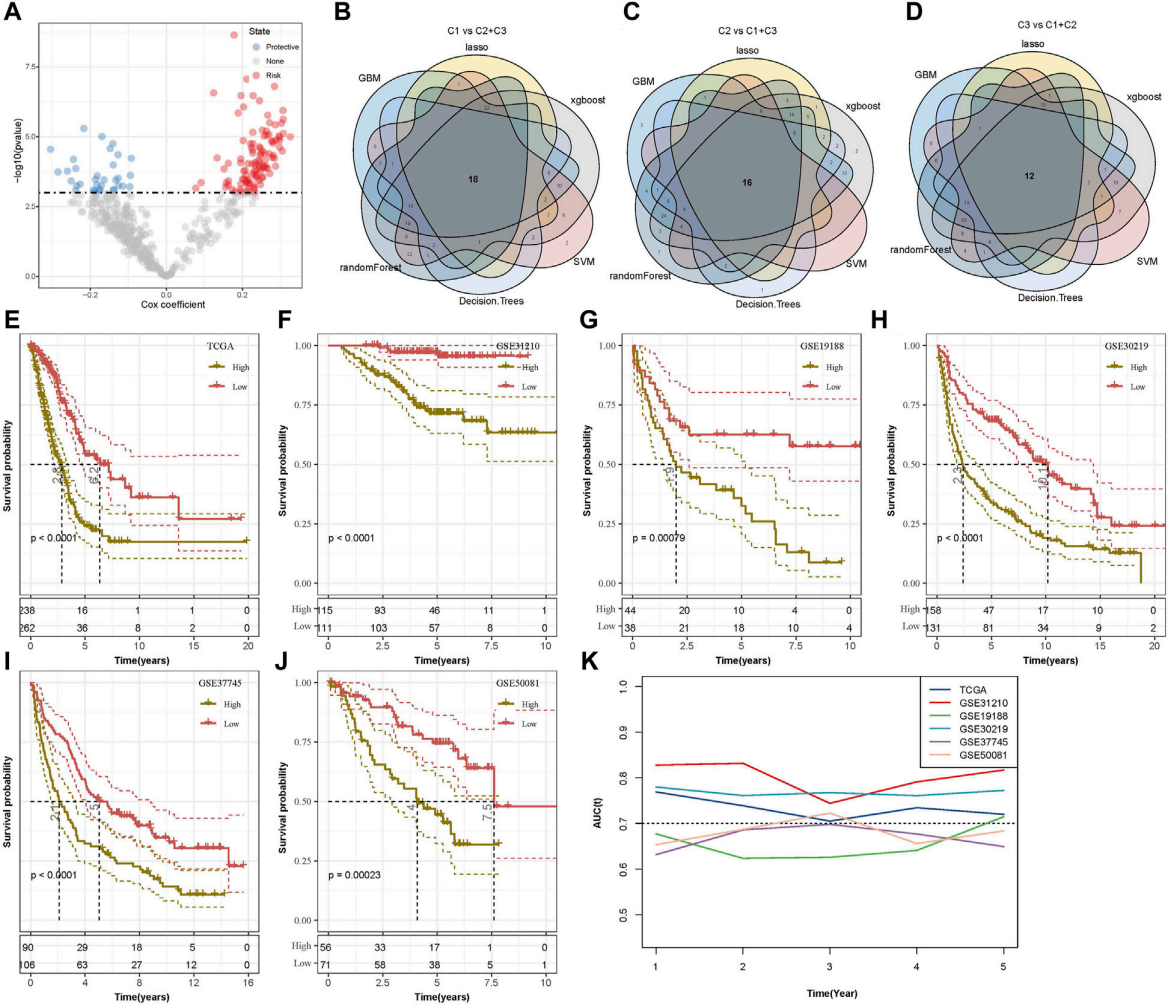
Key characteristic genes screening and K–M curves of six datasets. **(A)** Scatter plot of univariate Cox analysis of 493 genes associated with subtypes of FAM. **(B)** Venn diagram comparing C1 and C2 + C3 for characteristic gene screening of six algorithms. **(C)** Venn diagram comparing C2 and C1 + C3 for characteristic gene screening of six algorithms. **(D)** Venn diagram comparing C3 and C1 + C2 for characteristic gene screening of six algorithms. **(E)** K–M curve of the RiskScore model developed by the 12 genes in the TCGA cohort. **(F)** K–M curve of the RiskScore model developed by the 12 genes in the GSE31210 cohort. **(G)** K–M curve of the RiskScore model developed by the 12 genes in the GSE19188 cohort. **(H)** K–M curve of the RiskScore model developed by the 12 genes in the GSE30219 cohort. **(I)** K–M curve of the RiskScore model developed by the 12 genes in the GSE37745 cohort. **(J)** K–M curve of the RiskScore model developed by the 12 genes in the GSE50081 cohort. **(K)** Line graph of AUC for 1–5 years of RiskScore for six datasets.

Building upon these findings, univariate Cox analysis was used to perform prognostic analysis on 493 genes related to FAM subtypes, and 143 were screened to be prognostic genes relevant to LUAD (*p* < 0.001) (Figure 6A). Subsequently, we determined feature genes using six machine algorithms (including LASSO, GBM, random forest, SVN, XGBoost, and decision trees) for C1 and C2+C3, C2 and C1+C3, and C3 and C1+C2. Through the Venn diagram, we found a total of 18 characterized genes between C1 and C2+C3 (Figure 6B), a total of 16 genes between C2 and C1+C3 (Figure 6C), and a total of 12 genes between C3 and C1+C2 (Figure 6D). Further comparison and screening showed 34 important genes for subsequent studies. Finally, the number of important genes was reduced to 12 key genes (SLC2A1, PKP2, FAM83A, TCN1, MS4A1, CLIC6, UBE2S, RRM2, CDC45, IGF2BP1, ANGPTL4, and CD109) by the stepwise regression method.

The calculation formula is as follows:

RiskScore = −0.293*SLC2A1+0.145*PKP2+0.113*FAM83A+0.092*TCN1-0.142*MS4A1-0.081*CLIC6+0.24*UBE2S+0.217*RRM2-0.286*CDC45+0.162*IGF2BP1+0.093*ANGPTL4+0.112*CD109.

The RiskScore of the sample in TCGA was calculated by the abovementioned model formula, and high- and low-RiskScore groups were classified. The K–M curve results showed that the survival probability of the high-RiskScore group was lower (*p* < 0.0001, Figure 6E). At the same time, five sets of chip data (GSE31210, GSE19188, GSE30219, GSE37745, and GSE50081) were used to draw K–M curves, and the survival probability of the high-RiskScore group was still lower (Figures 6F–J). The AUC values of the 1-, 3-, and 5-year RiskScore of six datasets were observed, and it was found that the values of six datasets were all around 0.7 and that the AUC values of three datasets were consistently above 0.7, indicating that the model had a strong predictive performance (Figure 6K).

### 3.8 Testing the predictive performance of the RiskScore model combined with clinical characteristics

**FIGURE 7:**
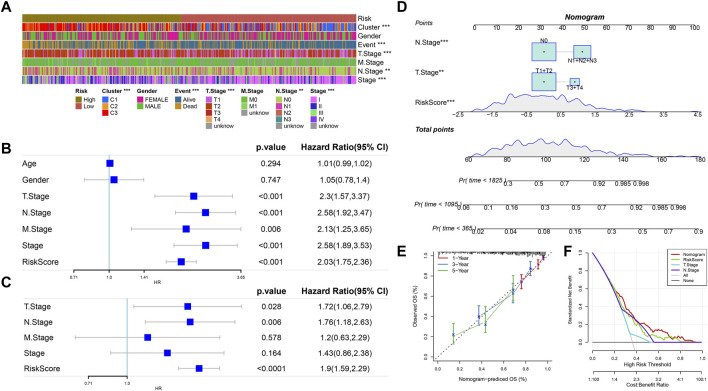
RiskScore combined with clinical characteristics to analyze the predictive performance of the model. **(A)** Heatmap of the distribution of clinical features in the high- and low-RiskScore group samples. **(B)** Univariate Cox analysis of clinical features and RiskScore. **(C)** Multivariate Cox analysis of clinical features and RiskScore (univariate prognostic correlation was selected here). **(D)** Nomogram model. **(E)** Calibration curves of the nomogram in 1, 3, and 5 years. **(F)** Decision curve of the nomogram.

Combined with the RiskScore, the distribution of samples with multiple clinical characteristics was presented in the form of a heatmap. The results showed that the distribution of five clinical characteristics (Cluster, Event, T Stage, N Stage, and Stage) was closely correlated with that of the RiskScore (Figure 7A). Meanwhile, the univariate Cox analysis between each clinical feature and the RiskScore showed that the *p*-values of T Stage, N Stage, Stage, and RiskScore were all less than 0.001. The multivariate Cox analysis showed that the *p*-values of RiskScore, T Stage, and N Stage were all less than 0.05. Therefore, T Stage, N Stage, and RiskScore were independent prognostic factors (Figures 7B, C). A nomogram was established by the abovementioned factors. According to the results, the RiskScore had the strongest survival prediction ability (Figure 7D). The slope and distance between all the calibration curves and standard ones were similar, which verified the nomogram’s prediction capability (Figure 7E). The benefit rates of the RiskScore and nomogram were significantly higher than the extremum curves, which proved that the nomogram and RiskScore had the greatest power for survival prediction (Figure 7F).

### 3.9 Potential regulatory pathways identified by the RiskScore model

**FIGURE 8:**
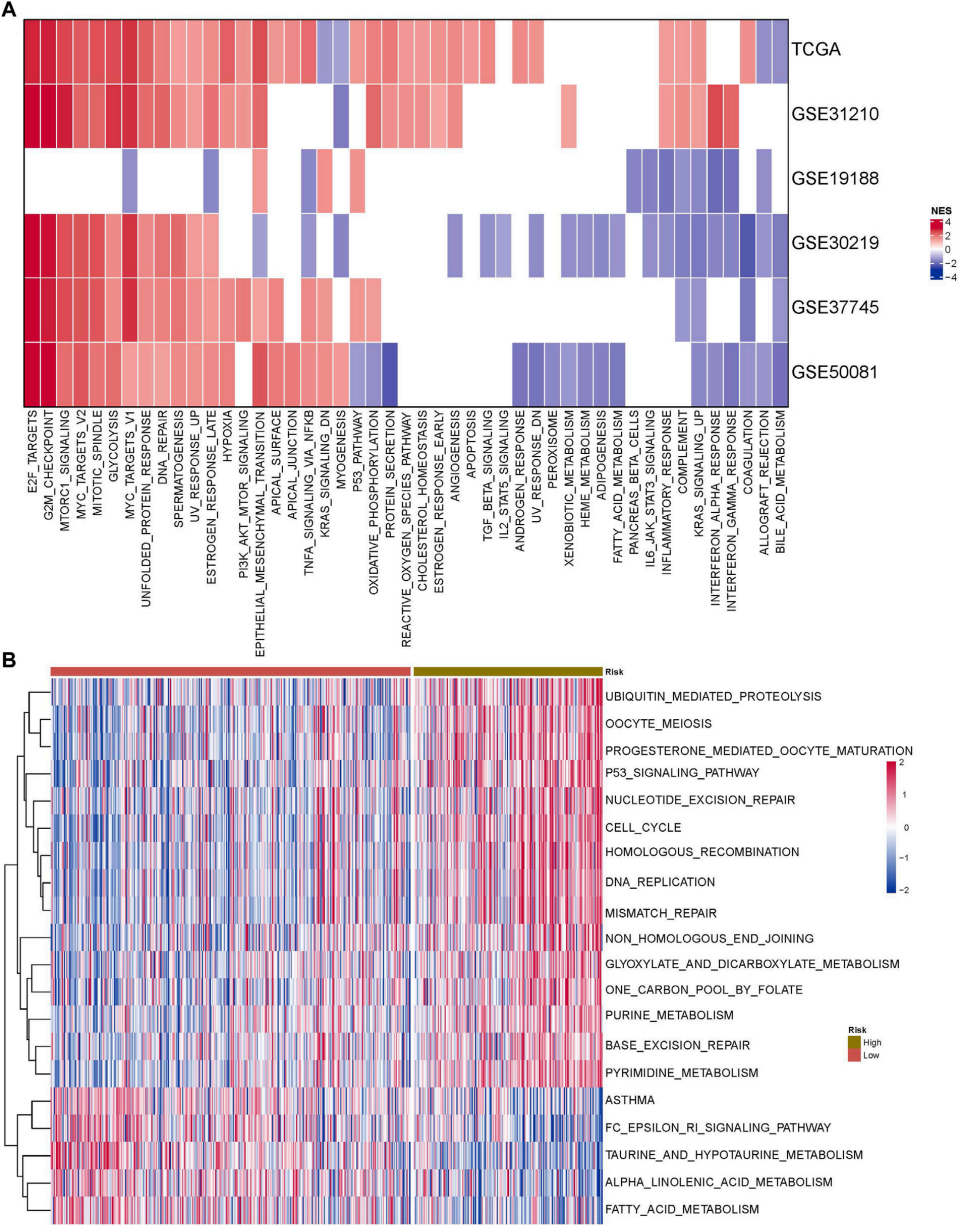
Combined with the RiskScore model, potential regulatory pathways were identified. **(A)** Pathway enrichment score heatmap obtained by six datasets in the HALLMARK gene set. **(B)** Heatmap of correlated pathway scores in the KEGG database TCGA cohort in high- and low-RiskScore groups.

The HALLMARK gene set was enriched in six datasets by GSEA, and it was found that E2F target and G2M checkpoint pathways had the highest scores, while bile acid metabolism and other pathways had lower scores. The FAM pathway score was also generally low (Figure 8A). We compared the predicted pathway scores of RiskScore groups in the TCGA cohort and observed that the scores of 15 pathways including ubiquitin-mediated proteolysis were higher in the high-RiskScore group, while the scores of five pathways including FAM were lower in the high-RiskScore group (Figure 8B).

### 3.10 Immune status and immunotherapy preference predicted by the RiskScore

**FIGURE 9:**
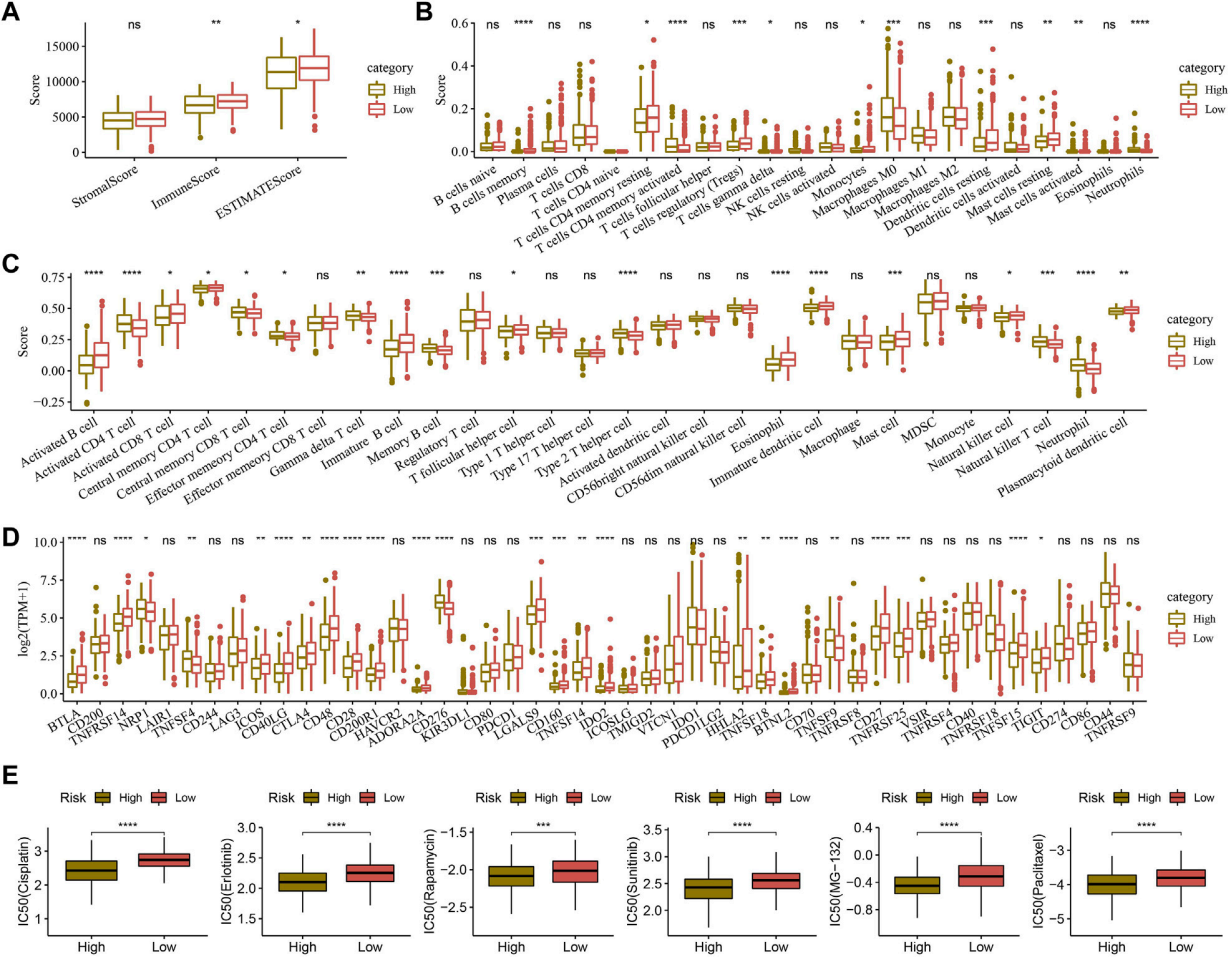
Comparison of high- and low-RiskScore groups with immune abnormalities. **(A)** Immune score was compared between low- and high-RiskScore groups. **(B)** In total, 22 immune cell scores were compared between low- and high-RiskScore groups. **(C)** In total, 28 immune cell scores were compared between low- and high-RiskScore groups. **(D)** Immune checkpoint gene expression was compared between low- and high-RiskScore groups. **(E)** Drug susceptibility was compared in low- and high-RiskScore groups. * *p* < 0.05, ** *p* < 0.01, *** *p* < 0.001, **** *p* < 0.0001, and ns represents *p* > 0.05.

The ImmuneScore and ESTIMATEScore were higher in the high-RiskScore group compared to those in the low-RiskScore group (Figure 9A). Among the 22 immune cell scores predicted, 11 immune cell scores, such as T-cell CD4 memory resting, showed significant differences between the two RiskScore groups (Figure 9B). Among the 28 immune cell scores predicted, 18 immune cell scores, such as activated CD4 T cells, showed differences between the two RiskScore groups (Figure 9C). The expression of 48 different immune checkpoint genes was compared between the two RiskScore groups, and higher expression of 19 immune checkpoint genes, such as BTLA, TNFRSF14, ICOS, and CD48, was found in the low-RiskScore group (Figure 9D).

Cisplatin, erlotinib, rapamycin, sunitinib, MG-132, and paclitaxel were all found to be more sensitive to the high-RiskScore group, suggesting that patients in high-RiskScore groups might respond better to these drugs (Figure 9E).

## 4 Discussion

Lung cancer is one of the most deadly malignant tumors worldwide (Wang et al., 2022b). The metabolic reprogramming of cancer cells, particularly the modification of FAM, is firmly connected with tumor growth (Maan et al., 2018). Abnormal FAM is associated with the growth, differentiation, and metastasis of LUAD cells. Acetyl-coA carboxylase 2 (ACC2) is a key FAM enzyme. Fei-Yuan Yu et al. found that ACC2 is low-expressed in tumor cells, and its expression is negatively correlated with tumor progression (Yu et al., 2022). FASN is a homodimeric multienzymatic protein that inhibits and blocks the adipogenic pathway and hinders fatty acid synthesis. This causes apoptosis in tumor cells to overexpress FASN without affecting non-malignant cells (Relat et al., 2012). Recent studies showed that FASN expression is upregulated and overactivated in LUAD, which may be related to the progression of LUAD (Relat et al., 2012). Drug targeting FAM pathways in LUAD has been designed. For example, AZ12756122, a novel FASN inhibitor, can induce cell apoptosis, downregulate FASN expression and activity, and reduce EGFR and Akt/mTOR pathway activation (Polonio-Alcalá et al., 2022). Although the relationship between the gene expression of FAM pathways and the prognosis of LUAD has been explored, this study introduced a variety of machine learning analysis algorithms to more comprehensively analyze the genetic characteristics of FAM pathways (Ganggayah et al., 2019), which can help establish a more effective risk prediction model for LUAD based on the FAM pathway genes.

Using LUAD data from the TCGA dataset, GEO dataset, and FAM-related gene sets obtained by KEGG analysis, we found that LUAD had lower scores of FAM-related pathways. Molecular subtypes were classified using the genes related to FAM pathways, and six machine learning methods were applied to select key genes related to the three LUAD subtypes. A total of 12 key genes (SLC2A1, PKP2, FAM83A, TCN1, MS4A1, CLIC6, UBE2S, RRM2, CDC45, IGF2BP1, ANGPTL4, and CD109) were determined to be closely related to LUAD prognosis. The downregulation of SLC2A1-AS1 can inhibit LUAD cell growth and expansion, and its overexpression increases tumor cell proliferation and differentiation. PKP2 promotes the growth, division, and migration of cancer cells through activating the EGFR signaling pathway in LUAD cells (Hao et al., 2019). FAM83A-AS1 knockdown can suppress the proliferation of LUAD cells, can inhibit the expression of HIF-1α and glycolytic genes, and also plays a role in FAM (Chen et al., 2022b). High expression of TCN1, a vitamin B12-binding protein, is positively associated with cancer aggressiveness and a poor prognosis (Li et al., 2022c). The expression level of MS4A1 in colorectal cancer is positively correlated with patients’ prognosis. CLIC6 is upregulated in most obese patients with endometrial cancer (López-Ozuna et al., 2021; Mudd et al., 2021). Mengjun Zhang et al. observed that UBE2S can promote PI3K or mTOR signaling pathway, block the regulation of cell cycle, inhibit cell apoptosis, and promote the proliferation, migration, and prognosis of ovarian cancer (Zhang et al., 2022b). RRM2 is upregulated in LUAD, and high RRM2 expression is associated with a poorer survival and lower immune infiltration (Ma et al., 2020). In addition, Zhou et al. demonstrated that RRM2 is overexpressed in the cell lines and clinical samples of bladder cancer and that blocking RRM2 inhibits the growth and proliferation of cancer cells (Zhou et al., 2022). CDC45, a key protein involved in the initiation of DNA replication, is upregulated in many cancers, and its expression is significantly negatively correlated with patient prognosis (Lu et al., 2022c). JinFeng Liu et al. found that IGF2BP1 is significantly abnormally expressed in LUAD samples. Moreover, ANGPTL4 is also significantly upregulated in LUAD samples, which are all closely related to the development and a poor prognosis of LUAD (Liu et al., 2022b; Yang et al., 2022). Tetsuro Taki et al. demonstrated the biological significance of regulating the TGF-β signal in the cancer cell matrix through the correlation verification of CD109 and LTBP1, and they indicated that the expression level of CD109 plays an important role in promoting the proliferation and diffusion of LUAD cells (Taki et al., 2020). Furthermore, Lee et al. demonstrated that C109 expression is correlated with the invasiveness and metastasis of LUAD. They observed that CD109 expression is mechanistically mediated by binding to EGFR to regulate AKT/mTOR signaling (Lee et al., 2020). Therefore, in this study, the selected FAM-related genes may all be involved in the progression of LUAD and can serve as biomarkers for the clinical diagnosis and treatment of cancer. Therefore, the 12 key genes were used to construct a RiskScore model, laying a foundation for the survival prediction and further study of LUAD.

LUAD tissues had a lower score of the FAM-related pathway compared to para-cancer tissues. This also indicated that abnormal FAM was involved in the progression and prognosis of LUAD, which is consistent with the characterization results of FAM in LUAD by Wang et al. (2022a). As a new treatment method, immunotherapy has become an effective strategy to treat cancers (Riley et al., 2019). The level of immune cell infiltration in the tumor microenvironment has also been used as an important indicator for assessing lung cancer (Liu et al., 2021c). There are many studies investigating the effect of FAM on immunotherapy in various cancers (Bleve et al., 2020). The differential expression of FASN is closely correlated with immune cell infiltration, and patients with a low expression of FASN have active response to immune checkpoint inhibitor treatment (Xiong et al., 2022). The analysis and comparison of various tumor-related studies showed that the upregulated FASN gene expression and activity is negatively correlated with tumor immune infiltration. The methylation of the FASN promoter in DNA can be used to serve as a new biomarker for cancer (Zhang et al., 2022c). Hence, a better understanding of the correlation between FAM and the immunological signature of the tumor microenvironment could facilitate the identification of new therapeutic targets for improving clinical cancer therapies.

However, this study also had certain limitations. The tumor and gene sample data collected were all from the database with a small number of samples, which demanded further *in vivo* or *in vitro* validation experiments to verify the predictive performance of the prognostic model. At the same time, the specific mechanism of abnormal FAM in LUAD was not clearly studied, and its interactions and regulatory mechanisms should be explored in depth.

## 5 Conclusion

In summary, this study determined 12 key genes (SLC2A1, PKP2, FAM83A, TCN1, MS4A1, CLIC6, UBE2S, RRM2, CDC45, IGF2BP1, ANGPTL4, and CD109) using six machine learning methods. A RiskScore model was constructed based on the 12 key genes mentioned previously. The model can accurately predict the survival of LUAD patients. We demonstrated that a specific model based on FAM could provide significant benefits for the precision treatment of LUAD and was effective in improving the prediction of patients’ prognoses.

## Data Availability

The datasets presented in this study can be found in online repositories. The names of the repository/repositories and accession number(s) can be found in the article/Supplementary Material.

## Abbreviations

ACC2, acetyl-coA carboxylase 2; BP, biological process; CC, cellular component; CDF, cumulative distribution function; CNV, copy number variant; DCA, decision curve analysis; FABP5, fatty acid-binding protein 5; FAM, fatty acid metabolism; FASN, fatty acid synthase; GEO, Gene Expression Omnibus; GSEA, gene set enrichment analysis; GO, Gene Ontology; KEGG, Kyoto Encyclopedia of Genes and Genomes; KM, Kaplan–Meier; LUAD, lung adenocarcinoma; MF, molecular function; SNV, single nucleotide variant; ssGSEA, single sample gene set enrichment analysis; and TCGA, The Cancer Genome Atlas.
